# Ultrasensitive and Fast Diagnostics of Viable *Listeria* Cells by CBD Magnetic Separation Combined with A511::*luxAB* Detection

**DOI:** 10.3390/v10110626

**Published:** 2018-11-13

**Authors:** Jan W. Kretzer, Mathias Schmelcher, Martin J. Loessner

**Affiliations:** Institute of Food, Nutrition and Health, ETH Zurich, Schmelzbergstrasse 7, 8092 Zurich, Switzerland; Jan.Kretzer@reg-ob.bayern.de (J.W.K.); mathias.schmelcher@hest.ethz.ch (M.S.)

**Keywords:** bacteriophage, diagnostics, *Listeria monocytogenes*, endolysin, magnetic separation, reporter phage

## Abstract

The genus *Listeria* includes foodborne pathogens that cause life-threatening infections in those at risk, and sensitive and specific methods for detection of these bacteria are needed. Based on their unrivaled host specificity and ability to discriminate viable cells, bacteriophages represent an ideal toolbox for the development of such methods. Here, the authors describe an ultrasensitive diagnostic protocol for *Listeria* by combining two phage-based strategies: (1) specific capture and concentration of target cells by magnetic separation, harnessing cell wall-binding domains from *Listeria* phage endolysins (CBD-MS); and (2) highly sensitive detection using an adaptation of the A511::*luxAB* bioluminescent reporter phage assay in a microwell plate format. The combined assay enabled direct detection of approximately 100 bacteria per ml of pure culture with genus-level specificity in less than 6 h. For contaminated foods, the procedure included a 16 h selective enrichment step, followed by CBD-MS separation and A511::*luxAB* detection. It was able to consistently detect extremely low numbers (0.1 to 1.0 cfu/g) of viable *Listeria* cells, in a total assay time of less than 22 h. These results demonstrate the superiority of this phage-based assay to standard culture-based diagnostic protocols for the detection of viable bacteria, with respect to both sensitivity and speed.

## 1. Introduction

The opportunistic human pathogen *Listeria monocytogenes* is exclusively transmitted via contaminated food, and poses a serious threat to immunocompromised individuals, children, and the elderly. Listeriosis is a disease which can cause severe symptoms such as meningitis, septicemia, and, in pregnant women, spontaneous abortion [1]. Although the incidence of listeriosis is relatively low, it is still of major concern due to reported average mortality rates of up to 30% [1,2,3]. As *L. monocytogenes* can multiply at refrigeration temperatures, even low contamination levels are considered a potential risk. Since foods that harbor *Listeria* generally feature a highly abundant and diverse background flora, highly sensitive diagnostic protocols are required. Standard culture-based methods for the detection of *Listeria* in foods such as the International Organization for Standardization (ISO) standard plating method (ISO 11290-1:2017) are still considered as the gold standard; however, they require a minimum of 96 h to obtain preliminary results. This emphasizes the need for alternative rapid screening and diagnostic protocols. A number of culture-independent detection methods for bacterial pathogens have been described, including polymerase chain reaction (PCR)-based protocols, mass spectrometry (MS), and immunological techniques. However, each of these methods has important drawbacks, such as the necessity for lengthy pre-enrichment procedures, the inability to distinguish between live and dead cells (PCR), the requirement for expensive devices (MS), or a lack in sensitivity and/or specificity (immunological assays) [4,5,6]. Over the past two decades, bacteriophages have emerged as ideal tools for bacterial diagnostics, particularly due to their extraordinary specificity for their target cells, their robustness and inexpensive production, and their ability to selectively detect viable cells [7]. A unique approach for the rapid detection of viable *Listeria* is the luciferase reporter bacteriophage A511::*luxAB* [8]. This derivative of the broad-host-range virulent *Listeria* phage A511 was constructed by inserting a fused *luxAB* gene from *Vibrio harveyi* downstream of the strongly expressed major capsid protein gene of the phage [8]. Upon infection of *Listeria* cells present in a given sample, a measurable bioluminescence signal is produced within approximately 60–150 min. A511::*luxAB* was then used for the detection of *L. monocytogenes* from artificially contaminated food and environmental samples [9]. With a total assay time of 24 to 48 h, the method was significantly faster than the standard plating protocols, while featuring similar sensitivity. However, the original assay format was somewhat impractical due to the use of single large plastic tubes with all manual handling, and could not be automated. In addition, the lower detection limit of approximately 1 × 10^3^ cfu/mL did not meet the stringent requirements in food safety testing.

The aims of this study were to adapt the reporter phage assay to a 96-well microwell plate format to enable high throughput screening of samples; to enhance the luminescent light signal by the addition of sodium azide (NaN_3_); and to combine the assay with upstream bead-based magnetic separation utilizing cell wall-binding domains from endolysins specific for *Listeria* spp. (CBD-MS) [6] in an effort to improve detection sensitivity and reduce the overall assay time. The functionalized magnetic beads allow rapid and efficient capturing and magnetic separation of *Listeria* cells from contaminated food samples, with genus-level specificity and high target cell affinity.

## 2. Materials and Methods

### 2.1. Bacteria and Culture Conditions

All bacteria used in this study were taken from the Weihenstephan *Listeria* collection (WSLC). *L. monocytogenes* strains EGDe (serovar 1/2 a), WSLC 1001 (ATCC 19112) (sv 1/2c), WSLC 1685 (ScottA) (sv 4b), WSLC 2012 (ATCC 33091) (sv 6b), and *Listeria ivanovii* WSLC 3009 (sv 5) were grown in half-concentrated brain heart infusion medium (BHI ½) (Oxoid, Hampshire, UK), at 30 °C. Cultures were incubated for 16 h, diluted fivefold in fresh medium, incubated for another 2 h, and subsequently diluted in phosphate buffered saline (PBS) supplemented with Tween 20 (Sigma-Aldrich, Buchs, Switzerland) (PBST; 50 mM Na_2_HPO_4_; 120 mM NaCl; 0.1% Tween 20; pH 8.0) to the desired concentration of cells.

### 2.2. Production of CBD Fusion Proteins and Coating of Magnetic Beads

N-terminally 6xHis-tagged fusion proteins consisting of the green fluorescent protein (GFP) and cell wall-binding domains (CBDs) from bacteriophage endolysins Ply118 and Ply500 (HGFP_CBD118 and HGFP_CBD500) were expressed in *Escherichia coli* and purified as previously described [6,10,11]. No glycerol was added to the purified protein preparations. Concentrations of purified proteins were adjusted to 2.5 mg/mL by the addition of PBS (50 mM Na_2_HPO_4_, 120 mM NaCl, pH 8.0). Protein preparations were stored at −20 °C until use. Paramagnetic polystyrene beads (Dynabeads M-270 Epoxy; Dynal, Oslo, Norway) were activated and coated with the purified CBDs as described previously [6].

### 2.3. Propagation of A511::luxAB

Bacteriophage A511::*luxAB* was propagated as described previously [9]. Phage concentration was adjusted to 1.0 × 10^9^ pfu/mL by the addition of SM buffer (SMB; 94 mM NaCl, 8 mM MgSO_4_ × 7H_2_O, 100 mM Tris, pH 7.4), and phage stocks were stored at 4 °C until use.

### 2.4. Downsizing the A511::luxAB Reporter Phage Assay from Single Tube to Microwell Format

For the adaptation of the A511::*luxAB* luciferase reporter phage assay [8,9] to microplate format, the optimum conditions concerning the bacteria/phage ratio, incubation time, and incubation temperature were determined. Experiments were carried out using pure cultures of *L. monocytogenes* ScottA, and the preparation of bacterial cells was as described above, with a slight modification: dilutions of subcultures were prepared using BHI (½) medium instead of PBST to ensure metabolic fitness of bacterial target cells during phage infection. Dilutions of subcultures with bacterial concentrations ranging from 3.0 × 10^3^ to 1.0 × 10^6^ cfu/mL were incubated with varying concentrations of phage particles ranging from 1.0 × 10^6^ to 3.0 × 10^8^ pfu/mL. The incubation temperature was varied in a range from 20 °C to 30 °C, whereas the incubation time was varied from 120 to 180 min. For each experiment, 200 µL of bacterial suspensions were mixed with 20 µL of phage suspensions in individual wells of a 96-well microwell plate (Black/White Isoplates (black frames with white well inserts), Perkin Elmer, Schwerzenbach, Switzerland). Measurements were performed using a multilabel microplate reader equipped with dual injectors (Viktor_3_, Perkin Elmer). Following preliminary experiments, the consecutive steps of the optimized measurement protocol were as follows: injection of 12 µL of a sodium azide (NaN_3_; Sigma-Aldrich) solution (1% in H_2_O, resulting in a final concentration of 7.6 mM), immediately followed by the injection of 12 µL of a nonanal (nonyl aldehyde, Sigma-Aldrich) solution (0.25% *v*/*v*, in 70% ethanol) (Loessner et al., 1996a). Measurement of light emission was performed using 10 s intervals for integration and calculation of relative light units (RLUs). The average value of RLUs per second was displayed as cps (counts per second).

### 2.5. Effect of NaN_3_

To determine the effect of NaN_3_ on the signal intensity in the A511::*luxAB* reporter phage assay, experiments with *L. monocytogenes* WSLC ScottA subcultures were carried out essentially as described above. Cell concentrations in samples ranged from 5.0 × 10^4^ to 5.0 × 10^6^ cfu/mL. The treatment of samples and subsequent measurements were performed at optimum conditions as determined before, with and without the use of NaN_3_ in the assay. Injection of 12 µL of a NaN_3_ solution (stock concentration: 1%, corresponding to 153.8 mM; final assay concentration: 7.6 mM) prior to the injection of 12 µL nonanal solution was then incorporated into the optimized protocol (see above).

### 2.6. Evaluation of the Combined CBD-MS/A511::luxAB Assay

One ml aliquots of *Listeria* cultures of the different strains tested (*L. monocytogenes* WSLC 1001, *Listeria innocua* WSLC 2012, *L. monocytogenes* ScottA, *L. monocytogenes* EGDe, and *L. ivanovii* WSLC 3009) containing approximately 1.0 × 10^2^–1.0 × 10^5^ cfu/mL, were mixed with 20 µL of CBD118-coated beads and 20 µL of CBD500-coated beads (2 × 10^7^ beads in total). The magnetic separation procedure was performed as previously described [6]. In brief, samples were incubated in an overhead rotator (NeoLab, Heidelberg, Germany) at 10 rpm for 40 min, and subsequently magnetic beads were separated using a MPC magnetic tube holder (Dynal, Oslo, Norway). Following careful removal of the supernatant, beads were washed with 1 mL of PBST, separated again, and finally resuspended in 200 µL of BHI (½). The bead suspension with captured bacterial cells was incubated in a horizontal shaker (Kühner, Birsfelden, Switzerland) at 37 °C and 180 rpm for 180 min and subsequently mixed with 20 µL of A511::*luxAB* to yield a final assay concentration of 1.0 × 10^8^ pfu/mL, followed by incubation at 24 °C for 140 min. Since preliminary testing indicated that the presence of beads could interfere with light emission, beads were magnetically separated as described above, and supernatants were collected and transferred to a microwell plate. To determine background luminescence, negative control samples containing either bacteriophages (1.0 × 10^8^ pfu/mL) but no *Listeria* cells, or *Listeria* cells (1.0 × 10^6^ cfu/mL) but no phage, were included in the experiments. All experiments were carried out in triplicate. The exact numbers of cells in each experiment used were determined by triplicate plating of 100 µL aliquots of appropriate dilutions of *Listeria* subcultures on BHI agar, and subsequent incubation at 37 °C for 16 h.

### 2.7. Testing Artificially Contaminated Foods

A set of nine different food items, including sliced iceberg lettuce, chocolate milk, mozzarella cheese, Swiss “Vacherin” red smear soft cheese, Swiss “Tomme” white mold soft cheese, ready-to-eat shrimp, minced meat, smoked salmon, and smoked turkey breast, was used in these experiments. All food samples were purchased at local retailers. First, each sample was aseptically divided into portions of 25 g each in a laminar flow hood. While one portion of each food was tested for the presence of *Listeria* (i.e., natural contamination) by standard procedures, all others were packed into sterile polypropylene plastic bags and immediately frozen at −80 °C. Samples that were free of *Listeria* were used for artificial contamination. Samples were thawed and inoculated with 1 mL each of diluted subcultures of either *L. monocytogenes* EGDe (serovar 1/2 a) or *L. monocytogenes* ScottA (serovar 4b) to obtain initial contamination levels ranging from 0.1 to 100 cfu/g food. In the case of solid foods, the inoculum was distributed on the surface of the sample by carefully massaging the bag. Exact initial contamination levels of food samples were determined by triplicate plating of 100 µL aliquots of appropriate dilutions of subcultures used for contamination. To determine the background, blank samples were prepared by adding 1 mL of PBST containing no *Listeria* cells to one sample of each food item.

All samples were then stored at 4 °C for 22–24 h, in order to simulate more realistic storage and sampling conditions. The authors did not observe any growth of the spiked *Listeria* cells during this period, which confirms other studies that have reported no significant increase in *L. monocytogenes* viable counts during initial storage at 4 °C for 24 h [12,13]. Subsequently, the 25 g food samples were homogenized with 50 mL of citrate buffer (for dairy products; 58 mM tri-sodium citrate dihydrate in H_2_O_dd_, pH 7.5) or PBS (other products) using a stomacher laboratory blender (Seward, West Sussex, UK). Samples were subjected to 16 h of selective enrichment in 175 mL tryptic soy broth (TSB, pH 7.5; Biolife, Milan, Italy) containing 12 mg/L acriflavine, 52 mg/L nalidixic acid and 64 mg/L cycloheximide (all from Sigma-Aldrich) (IDF 143A:1995).

Detection of *Listeria* spp. in enrichment cultures was performed essentially as described above. However, 100 µL of 10× PBST (tenfold concentrated) was added to each 1 mL sample taken from the enrichment cultures to adjust the pH values. Individual samples of each food item, contaminated with different numbers of cells of either *L. monocytogenes* EGDe or ScottA, were prepared. Analysis of each sample was carried out in duplicate.

### 2.8. Statistical Analysis

One-way analysis of variance (ANOVA) with a post hoc Dunnett’s multiple comparisons test was applied for comparison of log-transformed cps values with the respective control values in the combined CBD-MS/A511::*luxAB* assay. Two-way ANOVA followed by Sidak’s multiple comparisons test was used for comparing mean normalized cps values from reporter phage assays with and without the use of NaN_3_.

## 3. Results

### 3.1. Adaption of the Luciferase Reporter Phage Assay to Microwell Plates

**Figure 1:**
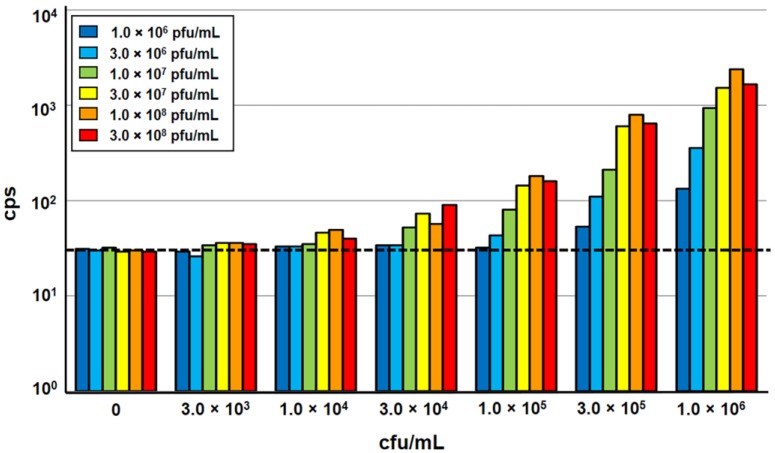
Evaluation of optimum bacteria:phage ratios in a 96-well microplate-based A511::*luxAB* assay. Increasing concentrations of *Listeria monocytogenes* ScottA (3.0 × 10^3^ to 1.0 × 10^6^ cfu/mL) were incubated with luciferase reporter phage A511::*luxAB* (1.0 × 10^6^ to 3.0 × 10^8^ pfu/mL). Bacteria were exposed to phage in 200 µL of half-concentrated brain heart infusion medium (BHI ½) in 96-well plates, and incubated at 24 °C for 140 min. Bioluminescence measurements were performed over a total period of 10 s, and are reported as counts per second (cps). The dotted horizontal line indicates the level of background light emission (noise) of the assay, determined in the negative control sample (without phage).

In order to allow higher throughput processing of samples, the A511::*luxAB* reporter phage assay, developed for use in a single tube luminometer [8,9], was adapted to a 96-well microplate format for processing in a semi-automated multi-label microplate reader equipped with programmable injectors. The different types of microwell plates initially tested yielded very different signal-to-noise ratios; the best results were obtained using black frame plates with white well inserts. Next, the impact of sample volume, bacteria-phage ratio, incubation temperature, and incubation time on light signal emission was determined. The results obtained with the reduced sample volumes (200 µL) in the microplate format only slightly differed from those measured in standard single tube-based measurements using 1 mL samples [8,9]. An incubation time of 140 min yielded the highest signal intensity, which confirmed that phage infection and luciferase-catalyzed light emission are largely independent on the test volume and luminescence detector. An increase of incubation temperature from 20 °C to 24 °C yielded a slightly higher signal intensity, without affecting the stability of the heat-sensitive LuxAB fusion protein synthesized in phage-infected target cells [6,8]. The variation of the phage concentration in the assay ratio revealed that 1.0 × 10^8^ pfu/mL is sufficient for the infection of *Listeria* target cells at variable concentrations up to 1.0 × 10^6^ cfu/mL. With the addition of more phage, no increase in signal intensity could be achieved, whereas lower concentrations resulted in a decrease in signal intensity (Figure 1).

### 3.2. Sodium Azide Enhances the Bioluminescence Signal

**Figure 2:**
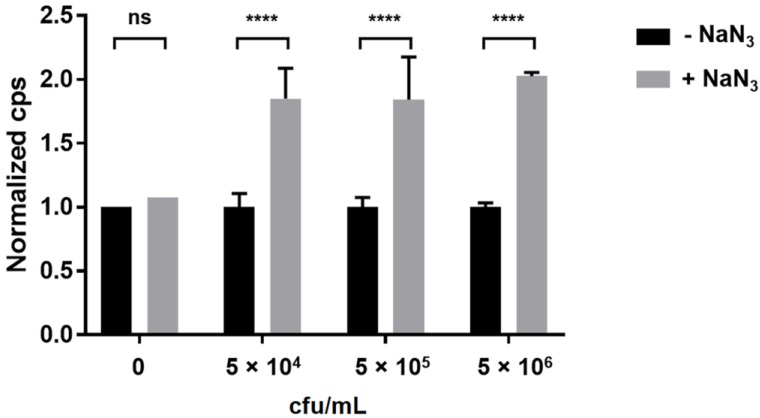
Effect of sodium azide (NaN_3_) addition on bioluminescent signal intensity. Different concentrations of *Listeria monocytogenes* ScottA cells ranging from 5.0 × 10^4^ to 5.0 × 10^6^ cfu/mL were infected with 1.0 × 10^8^ pfu/mL of A511::*luxAB*. Bacteria were incubated with phages in 200 µL of BHI ½ in microwell plates at 24 °C for 140 min. Measurements were performed in parallel with identical samples with and without the addition of 7.6 mM NaN_3_ as signal enhancer. For each bacterial concentration, bioluminescence signals have been normalized to the control sample (without NaN_3_). Asterisks indicate significant differences between values obtained with and without NaN_3_. ****, *p* < 0.0001; ns, non-significant.

The azide anion (N_3_^−^) from NaN_3_ inhibits the enzyme cytochrome oxidase in the respiratory chain [14,15]. This inhibition leads to a shift in the equilibrium of the reaction FMN → FMNH_2_ to its reduced form FMNH_2_. Here, FMNH_2_ is a co-factor required for the luciferase-driven oxidation of the aldehyde substrate in the A511::*luxAB* assay [8], and a limiting factor of the reaction. Therefore, an increase in FMNH_2_ concentration should result in increased light emission. To test this hypothesis, the effect of NaN_3_ on light emission from the reporter phage assay was evaluated. Following several rounds of optimization, the authors found that the injection of 7.6 mM NaN_3_ immediately prior to the injection of the nonanal luciferase aldehyde substrate significantly (*p* < 0.0001) increased the bioluminescence signal at all cell concentrations tested (Figure 2).

### 3.3. Combining CBD-MS Listeria Cell Separation with A511::luxAB Infection Yields Superior Sensitivity

**Figure 3:**
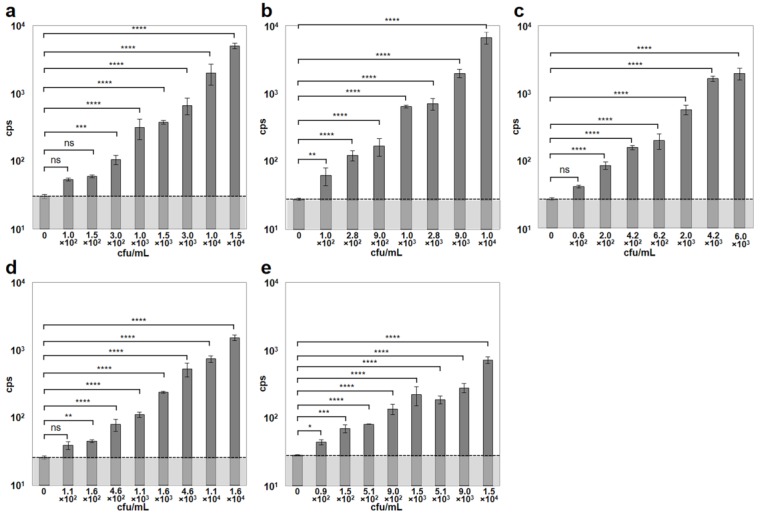
Detection of *Listeria* spp. strains employing the combined cell wall-binding domain-based magnetic separation (CBD-MS)/A511::*luxAB* assay. Dilutions of pure cultures of five different *Listeria* strains representing different serovars were used for evaluating the combined CBD-MS/A511::*luxAB* assay. (**a**) *L. monocytogenes* ScottA (serovar [SV] 4b); (**b**) *L. monocytogenes* EGDe (SV 1/2a); (**c**) *L. monocytogenes* WSLC 1001 (SV 1/2c); (**d**) *L. ivanovii* WSLC 3009 (SV 5); (**e**) *L. innocua* WSLC 2012 (SV 6b). The dotted horizontal bar indicates the level of background light emission (noise) of the assay, determined in the negative control sample (no bacteria). Asterisks indicate significant differences between values obtained from samples with bacteria and the respective control values. ****, *p* < 0.0001; ***, *p* < 0.001; **, *p* < 0.01; *, *p* < 0.05; ns, non-significant.

To further increase sensitivity and shorten the assay time, the authors combined the previously described CBD-MS method [6] with the microwell A511::*luxAB* assay. For this, paramagnetic microbeads coated with CBD118 and CBD500 from two different *Listeria* phages [11] were used to capture and separate *Listeria* cells of different strains, from dilute suspensions. The combined binding range of the two CBDs used here covered strains from all *Listeria* species and serovars [11]. Immediately following separation, the bacterial cells immobilized on the beads were resuspended in fresh media in the microplate wells, and allowed to recover for 3 h at 30 °C. Infection by the reporter phage and incubation at 24 °C were followed by bioluminescence measurement. The combined assay was able to reliably detect cell concentrations ≤ 3 × 10^2^ cfu/mL. The minimum bacterial concentrations yielding cps values significantly higher than the control were 3.0 × 10^2^ cfu/mL for *L. monocytogenes* ScottA, 1.0 × 10^2^ cfu/mL for *L. monocytogenes* EGDe, 2.0 × 10^2^ cfu/mL for *L. monocytogenes* WSLC 1001, 1.6 × 10^2^ cfu/mL for *L. ivanovii* WSLC 3009, and 0.9 × 10^2^ cfu/mL for *L. innocua* WSLC 2012, within a total time of 6 h or less (Figure 3). Generally, an increase in bacterial concentrations resulted in an increased luminescent signal intensity measured in the reporter phage assay. Negative control blank samples containing either no *Listeria* cells (Figure 3), or no bacteriophages yielded background signals (noise) ranging from 24 to 30 cps (mean = 28.1, SD = 1.83).

### 3.4. Detection of Listeria in Artificially Contaminated Food

**Figure 4:**
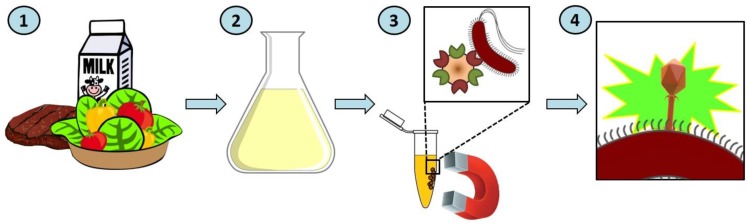
Schematic representation of the workflow of the combined CBD-MS/A511::*luxAB* assay. (**1**) food sample; (**2**) selective enrichment; (**3**) CBD-based magnetic separation; (**4**) A511::*luxAB* phage infection and generation of bioluminescent signal.

Finally, the authors evaluated the combined assay using nine different food items spiked with *Listeria* EGDe (serovar 1/2a) or ScottA (serovar 4b), at viable cell concentrations ranging from 0.1 to 100 cfu/g. The exact initial numbers added to the food samples was determined by serial plating on selective agar plates and yielded levels between 0.1 and 0.15 cfu/g for the lowest, and between 100 and 150 cfu/g for the highest contamination for both strains. The tested foods included chocolate milk, mozzarella cheese, sliced iceberg lettuce, ready-to-eat shrimp, minced meat, smoked salmon, smoked turkey breast, Vacherin-type red smear soft cheese, and Tomme-type white mold soft cheese. Following inoculation, the food samples were stored at 4 °C for 24 h to simulate more realistic conditions and give the bacteria time to accommodate to the environmental conditions. Samples of 25 g were then taken, and following a 16 h selective enrichment step, subjected to the combined CBD-MS/A511::*luxAB* assay, as depicted in Figure 4.

**Figure 5:**
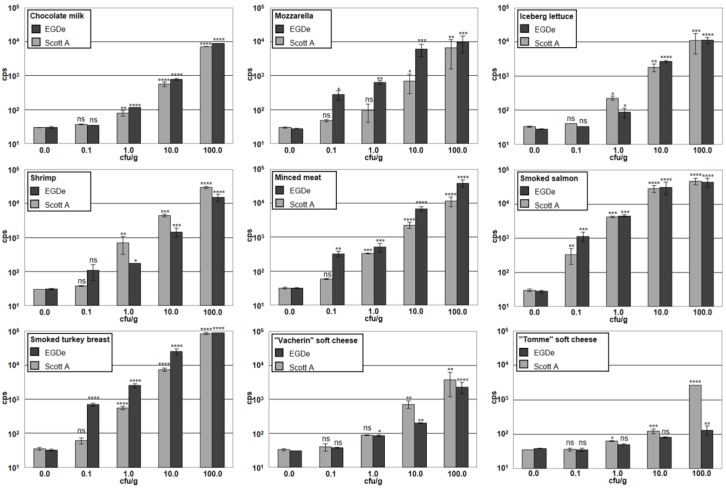
Detection of different concentrations of cells of *L. monocytogenes* ScottA and *L. monocytogenes* EGDe, in artificially contaminated food samples with the CBD-MS/A511::*luxAB* assay. Eight samples for each food item were spiked with bacteria of either ScottA or EGDe strains, at contamination levels of 0.1, 1.0, 10.0, or 100.0 cfu/g. Additionally, one blank sample for each food item was prepared by adding sterile buffer without bacteria. All samples were tested for the presence of *Listeria* spp. in parallel. Error bars represent SD from two replicates. Asterisks indicate significant differences between values obtained from samples contaminated with bacteria and the respective blank samples. ****, *p* < 0.0001; ***, *p* < 0.001; **, *p* < 0.01; *, *p* < 0.05; ns, non-significant.

The detection limits in these trials ranged from 0.1 cfu/g (for both strains in smoked salmon) to 100 cfu/g (for EGDe in “Tomme” soft cheese), and was between 0.1 and 1.0 cfu/g for most of the tested food items, except for the two soft cheeses, which yielded higher detection limits (Figure 5). The total assay time was 22 h. The data indicate that the signal intensity obtained from a specific sample nicely correlates with the number of target cells present, and also confirm that the different food items used in the experiments provide different growth conditions for the bacteria.

## 4. Discussion

The detection of pathogenic bacteria from potentially contaminated foods represents a major challenge for food producers, particularly when regulations allow only low bacterial concentrations or even require the complete absence of the pathogen (zero tolerance). The Draft Guidance for Industry on the “Control of *Listeria monocytogenes* in Ready-To-Eat Foods” by the U.S. Food and Drug Administration (FDA, 2017) recommends the implementation of listeriocidal process steps that ensure that *L. monocytogenes* is non-detectable in the final product. The current European Union legislation (Commission Regulation (EC) No 2073/2005) requires the absence of *Listeria monocytogenes* in 25 g of any food product that can support its growth until the end of shelf life. Therefore, any diagnostic protocols for *Listeria* must be highly sensitive and specific in order to prevent false-negative and false-positive results, respectively. Additionally, testing should ideally produce results within one day, to avoid recalls of products that have already reached the market by the time the test results are available. Further important factors include ease of use to allow application in the food industry and the ability to distinguish between viable and inactivated cells.

Standard selective plating methods like the International Dairy Federation (IDF) Standard 143A:1995 or the ISO 11290-1:2017 still represent the state of the art of *Listeria* detection in the food industry. Those selective enrichment/plating methods are specific and sensitive, and they can be applied in any standard laboratory. However, with any of these standard procedures, preliminary results will not be available within less than 96 h. Furthermore, insufficient recovery of stressed cells during food processing and slow or no growth on agars containing selective agents represent a possible disadvantage of these methods [16].

Over the past decades, many efforts have been made to improve the detection of *L. monocytogenes* in food by using culture-independent methods [17]. Nevertheless, almost all of these methods have certain drawbacks. Most currently available molecular detection methods are based on nucleic acid amplification, including conventional PCR [18], multiplex PCR [19,20], and quantitative real-time PCR [21,22]. Cost-effective alternative amplification methods that do not require thermal cycling have also been reported, and include loop-mediated isothermal amplification (LAMP) [23] and nucleic acid sequence-based amplification (NASBA) [24]. The popularity of PCR-based and similar methods likely lies in the potential of these techniques to detect very low concentrations of target DNA with maximum specificity, at reasonable cost, using readily available apparatus, with the possibility for automation. However, a major problem of PCR-based methods in food diagnostics is that the DNA polymerases are frequently inhibited by common food ingredients. To overcome this hurdle, some researchers have combined PCR with immunomagnetic separation (IMS) [25,26,27,28]. Using specific IMS, target cells may be separated from inhibitory substances and simultaneously concentrated, which increases the sensitivity of the assay. Although some of the studies reported detection of as low as 1.0 cfu/g from artificially contaminated foods [26], all PCR-based methods have another crucial disadvantage that cannot be eliminated: they cannot differentiate between inactivated and viable target cells. Even though primary contaminations of many raw food ingredients are usually eliminated by treatments such as temperature or high-pressure pasteurization, samples would still produce a positive signal.

Examples for other PCR-independent molecular diagnostic methods developed for *Listeria* include mass spectrometry techniques (MALDI-TOF) [29,30], assays that use biosensors measuring surface plasmon resonance [31,32], or methods based on flow cytometry [33,34]. Although these techniques may be of particular scientific interest, and some of them provide low detection limits, their application in the food industry has been limited due to the requirement for sophisticated and expensive equipment, and specifically trained operators.

Other approaches combined standard plating methods with antibody-based IMS or IMS-like procedures to improve sensitivity and reduce assay time [35,36,37,38]. Although a reduction of the required time down to 48 h could be achieved in these studies, and detection limits ranged from 1.0 cfu/25 g [38] to 1.0 cfu/g [39], the approaches have other disadvantages. A major problem of IMS is the lack of proper specificity of the antibodies used, and undesired cross-reactivity with other organisms than the target organism is quite frequent [36,37,39].

The authors addressed this problem by replacing antibodies with cell wall-binding domains (CBDs), which are recombinant high-affinity domains derived from *Listeria* phage endolysins. They work extremely well for the functionalization of paramagnetic beads, and for the subsequent magnetic capture and separation of *L. monocytogenes* from artificially and naturally contaminated food samples [6]. The CBDs generally feature high affinity and extreme specificity for their target cells, with no cross-reaction with other bacteria. This CBD-MS assay was shown to be superior compared to the standard plating protocols in both time requirement (48 h) and sensitivity, with relatively low detection limits for most of the food items tested. However, the assay still relied on surface plating following magnetic separation, which accounted for half of the total assay time. To further shorten the time required to obtain results, the authors employed in this study A511::*luxAB* reporter phage detection of the captured bacteria, including scaling to microplate format, and further optimizations such as optimized incubation temperature, phage concentration, and use of a light emission enhancer. Altogether, the combined CBD-MS/A511::*luxAB* assay consisted of four steps: (i) selective separation of *Listeria* cells from an enrichment culture or other sample by CBD-MS; (ii) washing and concentration of *Listeria* cells immobilized on beads, followed by enrichment in non-selective medium in order to improve the sensitivity of the assay and resuscitate injured cells; (iii) infection with A511::*luxAB*; and (iv) removal of magnetic beads and measurement of light emission in a semiautomatic plate reader, following injection of signal enhancer and luciferase substrate.

Using pure bacterial cultures, as few as 90 cfu/mL could be detected in as little as 6 h by the assay. When contaminated foods are examined, the assay includes a selective pre-enrichment step. This selective step is necessary because of the high number of organisms in the background flora of many food products, which would otherwise overgrow the *Listeria* cells. However, the selective pre-enrichment is significantly shorter than in the conventional culture-based protocols, and the authors’ method achieved a detection limit of 0.1 to 1.0 cfu/g in less than 24 h for most food items. This renders the assay significantly faster than the initial CBD-MS [6] and A511::*luxAB* reporter phage methods [9], with improved sensitivity.

Besides sensitivity and speed, the CBD-MS/A511::*luxAB* assay has another important advantage compared to magnetic-separation methods that use plating on selective agar as an end-point detection. Due to food processing, microorganisms present in food are often subjected to sublethal stresses. As these cells might be non-cultivable in selective media, they may remain undetected by the plating method. In this study, the incubation of *Listeria* immobilized and separated on CBD-coated beads serves as a resuscitation step and certainly improves detection of physiologically injured cells.

An important advantage of this bacteriophage-based assay is its extreme specificity, essentially eliminating undesired cross-reactivity with other bacterial genera by including three independent *Listeria*-specific selection steps: (i) selective enrichment for *Listeria* cells; (ii) CBD-MS using CBDs that have been demonstrated to be specific for *Listeria* [11]; and (iii) infection by a reporter phage based on the strictly genus-specific A511 phage [40]. It should be noted, however, that the assay as described here will not only signal the presence of *L. monocytogenes,* but it will also indicate other *Listeria* species sensitive to A511 infection. Yet, the authors believe that information on the presence of *Listeria* species is of great value, whereas testing for *L. monocytogenes* alone may not provide the full picture, that is, information on insufficient processing or potential contamination sources and routes is missing. Yet, if such specificity is desirable, a combination of the CBD-based enrichment and *L. monocytogenes*-specific PCR can also be used, as reported earlier [41].

A limitation of the assay is that A511::*luxAB* infects approximately 95% of all relevant *Listeria monocytogenes* strains of serovars 1/2 and 4 [40,42].Therefore, up to 5% of potential target strains may not be detected by this approach. However, it should be noted that available evidence suggests that the resistance of *Listeria* to phages such as A511 and others is due to loss of cell-wall associated carbohydrate decorations in their wall teichoic acids [43], and that these strains reveal a strongly attenuated virulence [44].

It is therefore concluded that, due to their unrivaled specificity for their hosts, genetically engineered reporter phages and phage-derived affinity proteins such as the CBDs represent very versatile and powerful tools for the detection of target host cells, including pathogenic bacteria. In this study, the authors combined two such phage-based techniques into one detection protocol, thereby harnessing the extraordinary specificity of bacteriophage in different ways. In terms of both sensitivity and time requirement, the assay seems superior to previous methods, including culture-based protocols.
